# Novel Sulfur Metabolites of Garlic Attenuate Cardiac Hypertrophy and Remodeling through Induction of Na^+^/K^+^-ATPase Expression

**DOI:** 10.3389/fphar.2017.00018

**Published:** 2017-01-30

**Authors:** Tarak N. Khatua, Roshan M. Borkar, Soheb A. Mohammed, Amit K. Dinda, R. Srinivas, Sanjay K. Banerjee

**Affiliations:** ^1^Division of Medicinal Chemistry and Pharmacology, Indian Institute of Chemical TechnologyHyderabad, India; ^2^Drug Discovery Research Center, Translational Health Science and Technology InstituteFaridabad, India; ^3^National Centre for Mass Spectrometry, Indian Institute of Chemical TechnologyHyderabad, India; ^4^Department of Pathology, All India Institute of Medical SciencesNew Delhi, India

**Keywords:** garlic, hypertrophy, Na^+^/K^+^-ATPase, isoproterenol, garlic-metabolites

## Abstract

Epidemiologic studies show an inverse correlation between garlic consumption and progression of cardiovascular disease. However, the molecular basis for the beneficial effect of garlic on the heart is not known. Therefore, the objective of the present study was to (1) investigate the effect of raw garlic on isoproterenol (Iso) induced cardiac hypertrophy (2) find the active metabolites of garlic responsible for the beneficial effect. Cardiac hypertrophy was induced in rats by subcutaneous single injection of Iso 5 mg kg^-1^ day^-1^ for 15 days and the effect of garlic (250 mg/kg/day orally) was evaluated. Garlic metabolites in *in vivo* were identified by LC/MS study. The effect of garlic and its metabolites were evaluated against hypertrophy in H9C2 cells. Garlic normalized cardiac oxidative stress after Iso administration. Cardiac pathology and mitochondrial enzyme activities were improved in hypertrophy heart after garlic administration. Decreased Na^+^/K^+^-ATPase protein level that observed in hypertrophy heart was increased after garlic administration. We identified three garlic metabolites in rat serum. To confirm the role of garlic metabolites on cardiac hypertrophy, Na^+^/K^+^-ATPase expression and intracellular calcium levels were measured after treating H9C2 cells with raw garlic and two of its active metabolites, allyl methyl sulfide and allyl methyl sulfoxide. Raw garlic and both metabolites increased Na^+^/K^+^-ATPase protein level and decreased intracellular calcium levels and cell size in Iso treated H9C2 cells. This antihypertrophic effect of garlic and its sulfur metabolites were lost in H9C2 cells in presence of Na^+^/K^+^-ATPase inhibitor. In conclusion, garlic and its active metabolites increased Na^+^/K^+^-ATPase in rat heart, and attenuated cardiac hypertrophy and associated remodeling. Our data suggest that identified new garlic metabolites may be useful for therapeutic intervention against cardiac hypertrophy.

## Introduction

Hypertrophy followed by heart failure is a major health problem of every nation. It is well recognized that causes of hypertrophy are multifactorial. The most common risk factors for cardiac hypertrophy are coronary heart disease, high blood pressure, diabetes and idiopathic cardiomyopathy. Cardiac hypertrophy is a cellular response and viewed as a compensatory response to biochemical stress and haemodynamic changes (Kang, 2006). Cardiac hypertrophy is a potent risk factor for the development of cardiac arrhythmias, diastolic dysfunction, congestive heart failure and death. Sustained pathological hypertrophy is deleterious and may leads to heart failure and death (Frey and Olson, 2003). It is also a risk factor for QT prolongation and cardiac sudden death. Therefore, it is very important to elucidate the molecular mechanism and potent remedy to prevent cardiac hypertrophy.

The intracellular factors that contribute to cardiac remodeling include oxidative stress, mitochondrial dysfunction, reactive oxygen species (ROS) generation and inflammation. One of the features of cardiac remodeling is a progressive impairment in mitochondrial function (Verdejo et al., 2012). The heart has a large number of mitochondria, which form a complex network under constant remodeling in order to sustain the high metabolic rate of cardiac cells when oxygen supply is threatened. High release of electrons from the electron transport chain leads to ROS generation and mitochondrial dysfunction (Turrens, 2003).

Along with mitochondrial dysfunction and ROS generation, expression of several signaling proteins altered that impaired the cardiac function (Takimoto and Kass, 2007). Na^+^/K^+^-ATPase is a major transporter for active ion transport across the sarcolemma and thus play an important role in controlling the electrical as well as the contractile function of the myocardium. It was revealed that reduction of Na^+^/K^+^-ATPase in the heart leads to contractile dysfunction, generation of arrhythmias and heart failure (Schwinger et al., 2003). Thus, augmentation or normalization of Na^+^/K^+^-ATPase level in the heart through nutritional or pharmacological agent might be responsible for attenuation of cardiac hypertrophy and remodeling.

Garlic, (*Allium sativum*) is a perennial herb and abundant with sulfur compounds. The role of garlic in reducing hyperlipidemia, hypertension, and platelet aggregation have been well established (Rahman and Lowe, 2006). Previously, garlic oil has been shown to prevent cardiac hypertrophy in hypercholesterol-fed hamster (Hsieh et al., 2014). The beneficial effect of garlic in heart might be through activation of nitric oxide pathway. The role of garlic to enhance nitric oxide production is well documented (Khatua et al., 2012, 2016). Nitric oxide plays a crucial role to modulate cardiac Na^+^/K^+^-ATPase activity (Pavlovic et al., 2013). However, no study has been addressed to find the effect of raw garlic and its metabolites on cardiac hypertrophy and Na^+^/K^+^-ATPase level in heart. Therefore, we aim to determine the effect of raw garlic and its metabolites on attenuation of cardiac hypertrophy *in vitro* and/or *in vivo* by modulating Na^+^/K^+^-ATPase level.

## Materials and Methods

### Garlic Homogenate

Garlic (*A. sativum*) bulbs were purchased from a local market. Cloves were peeled, sliced, ground into a paste and then suspended in distilled water. Rats were fed freshly prepared aqueous garlic homogenate every day through esophagus by oral gavage.

### Animals

All animal experiments were undertaken with the approval of Institutional Animal Ethics Committee of Indian Institute of Chemical Technology (IICT), Hyderabad. All experiments were performed in accordance with IICT guidelines and regulations.

### Experimental Protocol

#### Rat Model

Weight matched (200 g) male Sprague-Dawley rats were randomly divided into three groups of eight rats each. Experimental groups are described in **Table 1**. Control rats were fed normal saline daily for 15 days. Aqueous garlic homogenate was fed by oral gavage every day at a fixed time (10:00 AM) for 15 days at a dose of 250 mg/kg. Two test groups (Iso and Iso+Garlic) were given Iso subcutaneously every day at a fixed time (10:00 AM) for 15 days at a dose of 5 mg/kg/day. At the end of 15 days, 2 ml blood was aspirated from the left ventricle, collected in a heparinized vial, centrifuged at 3000 *g* for 10 min and the plasma was stored at -80°C for estimation of plasma LDH, SGOT, NO and H_2_S levels. At the end of 15 days, rats are sacrificed and hearts were stored at -80°C for estimation of all other tissue parameters.

**Table 1:** Experimental groups.

SL No	Groups	Description
1	Control	Subcutaneous injection of normal saline daily for 15 days.
2	Iso	Subcutaneous injection of Iso 5 mg kg^-1^ day^-1^ for 15 days.
3	Iso+Garlic	Oral administration of garlic homogenate 250 mg kg^-1^ day^-1^ for 15 days along with Iso as administered in Iso group.

#### Cell Culture

H9C2 cell line is derived from embryonic rat heart tissue and exhibits many morphological characteristics similar to those of immature embryonic cardiomyocytes but has preserved several elements of the electrical and hormonal signal pathway found in adult cardiac cells. Therefore, this cell line was used in the present study to induce hypertrophy (Hescheler et al., 1991). H9C2 cells were purchased from ATCC, USA and cultured using Dulbecco’s Modified Eagle Medium (DMEM) containing10% fetal bovine serum (FBS). Cells from passages 2–7 were used for all the experiments. Cells were seeded into a six-well plate and allowed to proliferate until 70–80% confluence. Cells were treated with Iso (25 μM) for 48 h and then treated with fresh aqueous garlic extract (0.25 mg/ml), AMS (100 nM/L) and AMSO (100 nM/L) for 24 h. Cell size, an important parameter of hypertrophy is measured. Protein was collected for western blot analysis of Na^+^/K^+^-ATPase. To find the role of Na^+^/K^+^-ATPase on hypertrophy in H9C2 cells, Digoxin (100 nM) (Ivana et al., 2012) was pre-treated along with Iso for 48 h followed by 24 h treatment of Garlic and its metabolites (AMS and AMSO). More details of this experiment were given in the Supplementary File.

### Measurement of Intracellular Calcium in H9C2 Treated Cells

After 70% confluency, H9C2 cells were treated with Iso, garlic and its metabolites. After 48 h of treatment, media was discarded and cells were loaded with 1 μM Fura 2-AM in phosphate buffer for 45 min at 37°C. Dye was removed and cells were washed with PBS two times. The cells were suspended in phosphate buffer and centrifuged. Then cell pellet was resuspended and lysed with RIPA buffer. Then fluorescence was measured at excitation spectra at 340/380 nm with emission at 510 nm. Intracellular free calcium was expressed in terms of percentage of fluorescence per microgram protein.

### Measurement of Garlic Metabolites by U-HPLC-MS Study

We used U-HPLC-MS method for separation of garlic metabolites. In brief, six adult male Sprague-Dawley rats were administered (250 mg/kg of garlic homogenate) orally for 15 days. The blood samples were collected at 15 min, 30 min, 1, 2, 4, 6, 8, and 10 h. Blood samples were pooled together to generate a single sample of matrix. Pooled samples were thoroughly vortex and centrifuged at 5000 rpm for 15 min at 4°C; and plasma was collected and stored at -20°C until analysis. Samples were prepared by simple protein precipitation method.

### Cardiac Hypertrophy

Daily subcutaneous single injection of Iso at a dose of 5 mg/kg body weight for 15 days creates cardiac hypertrophy in rats (Alderman and Harrison, 1971; Osadchii, 2007). In each group, the heart weight/body weight ratio and β MHC gene expression in heart were measured as indicators of cardiac hypertrophy.

### Serum Parameters

Plasma SGOT and LDH were estimated as markers of cardiac injury. SGOT, LDH activity and nitric oxide were estimated by according to the method described by (Khatua et al., 2012). Myocardial H_2_S concentration was measured from heart homogenate as described by Sojitra et al. (2012).

### Myocardial Conjugated Dienes Level and Antioxidant Status

Myocardial conjugated dienes is a measure for membrane lipid peroxidation and were estimated according to the method described by Devasagayam et al. (2003). Myocardial GSH content, SOD activities and myocardial catalase activity in heart homogenate were measured by the method as described (Gupta et al., 2013). Total antioxidant activity was measured by DPPH assay as described earlier (Khatua et al., 2012).

### Mitochondrial Enzyme Activity in Heart

Activities of mitochondrial enzymes, e.g., citrate synthase and β hydroxyacyl CoA dehydrogenase and cytochrome C oxidase were estimated to measure the extent of mitochondrial dysfunction. These enzymes were measured according to the method described by Civitarese et al. (2007).

### Immunoblot Analysis

Total protein extraction and immunoblotting were performed as described previously (Banerjee et al., 2009). Protein concentration was determined by Bradford method. An equal amount (40 μg) of protein was separated by sodium dodecyl sulfate polyacrylamide gel electrophoresis (SDS–PAGE). After electrophoresis, protein was transferred to PVDF membranes (Millipore, USA). The membranes were then blocked in Tris-buffered saline Tween-20 (TBS-T; 10 mM Tris, pH 7.5, 150 mM NaCl, 0.05% Tween-20) and 5% non-fat dry milk for 1 h, and subsequently washed and incubated with primary antibodies in TBST with 3% non-fat dry milk at 4°C for overnight. The following polyclonal antibody and titer was used: Na^+^/K^+^-ATPase (1:1,000, Cell Signaling Technology # 3010), After washing with TBS-T, membrane was incubated with Goat Anti-Rabbit IgG– HRP (1:4000 dilution, Cell Signaling Technology, # SC 2004) horseradish peroxidase conjugated secondary antibody with 2.5% non-fat dry milk at room temperature for 2 h. After washing with TBS-T immunoreactions were visualized with a chemiluminescence detection kit (Prod No- 34080, Super signal^®^ west Pico chemiluminescent substrate, Thermo Scientific). Then the blots were exposed to X- ray film (Hyperfilm ECL, GE Healthcare, USA) and developed. Gel stained with coomassie blue served as an equal loading control. Quantification of band intensity was performed using the Image J Software (NIH, Bethesda, MD, USA). For bar graph generation of Na^+^/K^+^-ATPase, the density of bands was obtained from two independent western blots runs under the same experimental conditions. Gel stained with coomassie blue served as an equal loading control.

### Gene Expression

Total RNA was extracted from the whole heart with Trizol (Sigma) and cDNA was prepared according to the method described by Banerjee et al. (2009). PCR was performed for collagen I, RPL32 and β-MHC mRNA expression. Rat collagen I forward primer (5′-ACGTCCTGGTGAAGTTGGTC-3′) and reverse primer (5′-CAGGGAAGCCTCTTTCTCCT-3′), RPL32 forward primer 5′-AGATTCAAGGGCCAGATCCT-3′ and reverse primer 5′-CGATGGCTTTTCGGTTCTTA-3′), β-MHC forward primer 5′-TGGAGCTGATGCACCTGTAG-3′ and reverse primer 5′- ACTTCGTCTCATTGGGGATG-3′, BAX forward primer 5′-TGCAGAGGATGATTGCGACT-3′ and reverse primer 5′-GATCAGCTCGGGCACTTTAG-3′, Caspase 3 forward primer 5′-AGGCCGACTTCCTGTATGCT-3′ and reverse primer 5′-TCCGGTTAACACGAGTGAGG-3′, and BAD forward primer 5′-AGTGAGCAGGAAGACGCTAG-3′ and reverse primer 5′-TAAGCTCCTCCTCCATCCCT-3′.

### Histopathology

Masson’s Trichrome is commonly used for staining collagen and detecting cardiac fibrosis. Heart tissue was fixed in 10% formalin, routinely processed and embedded in paraffin. Paraffin sections (3 μm) were cut on glass slides, stained with Masson’s Trichrome and examined under a light microscope to find myocardial fibrosis. Two sections of heart from each group were analyzed for histopathology in a blinded manner.

### Statistical Analysis

All values were expressed as mean ± SEM. Data were statistically analyzed using one-way ANOVA for multiple group comparison followed by Bonferroni *post hoc* test. Significance was set at *p* < 0.05.

## Results

### Garlic Treatment Attenuated Cardiac Hypertrophy in Rat Heart

**FIGURE 1:**
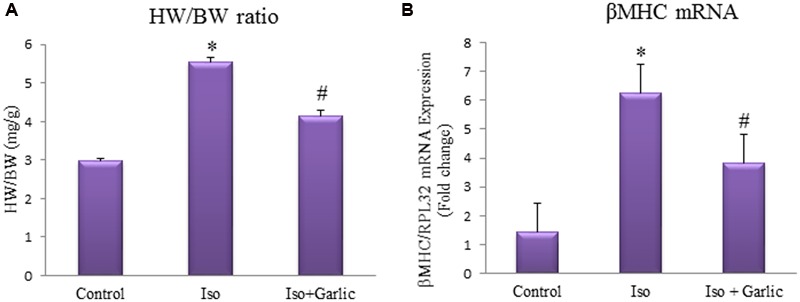
**Effect of garlic on Iso-induced cardiac hypertrophy. (A)** Heart weight and body weight ratio. **(B)** Expression of β-MHC mRNA. Data were shown as mean ± SEM (*N* = 6), ^∗^*p* < 0.05 vs. Control, ^#^*p* < 0.05 vs. Iso.

Heart weight/Body weight ratio and β-MHC gene expression were measured as markers of cardiac hypertrophy. These were significantly (*p* < 0.05) increased in Iso group compared to control group and significantly (*p* < 0.05) decreased in Iso+garlic group compared to Iso group (**Figures 1A,B**).

### Garlic Treatment Reduced Cardiac Injury Parameters and Normalized Serum NO and H_2_S Level

**FIGURE 2:**
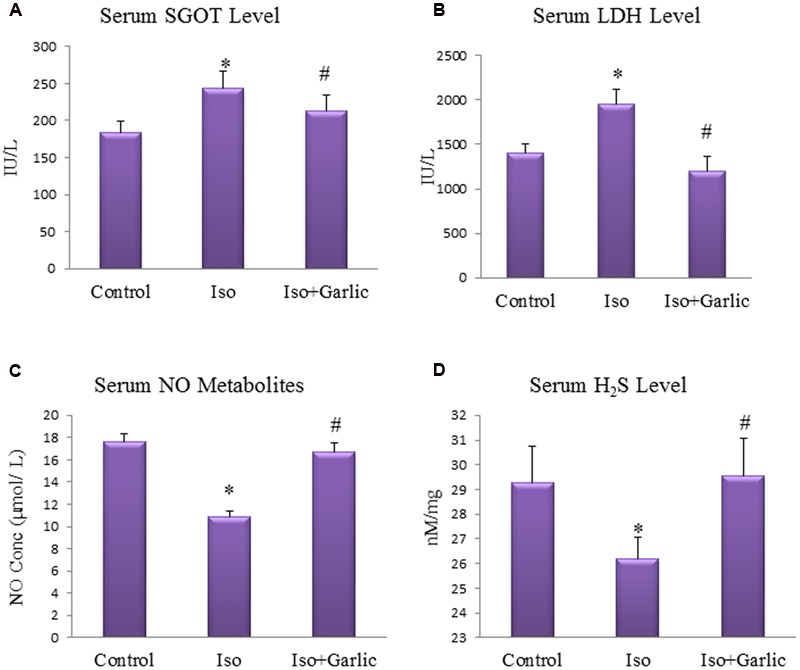
**Effect of garlic on cardiac injury parameters. (A)** Serum SGOT. **(B)** Serum LDH. **(C)** Serum nitric oxide. **(D)** Serum hydrogen sulfide. (*N* = 6). Data were shown as mean ± SEM, ^∗^*p* < 0.05 vs. Control, ^#^*p* < 0.05 vs. Iso.

Serum SGOT and LDH are two important serum markers for cardiac injury. A significant (*p* < 0.05) increase in both SGOT and LDH levels was observed in Iso group compared to control. However, a significant (*p* < 0.05) decrease in both SGOT and LDH levels was observed in Iso+garlic group in comparison to Iso group (**Figures 2A,B**).

On the other hand nitric oxide and hydrogen sulfide, two endogenous gaseous molecules of endothelial cells are protective indicators of the cardiac system. There were significant (*p* < 0.05) decrease in serum NO metabolites and hydrogen sulfide levels in Iso group compared to control group. However, a significant (*p* < 0.05) increase in serum NO metabolites and hydrogen sulfide levels was observed in Iso+garlic group compared to Iso group (**Figures 2C,D**).

### Garlic Treatment Attenuated Oxidative Stress in Hypertrophy Rat Heart

**FIGURE 3:**
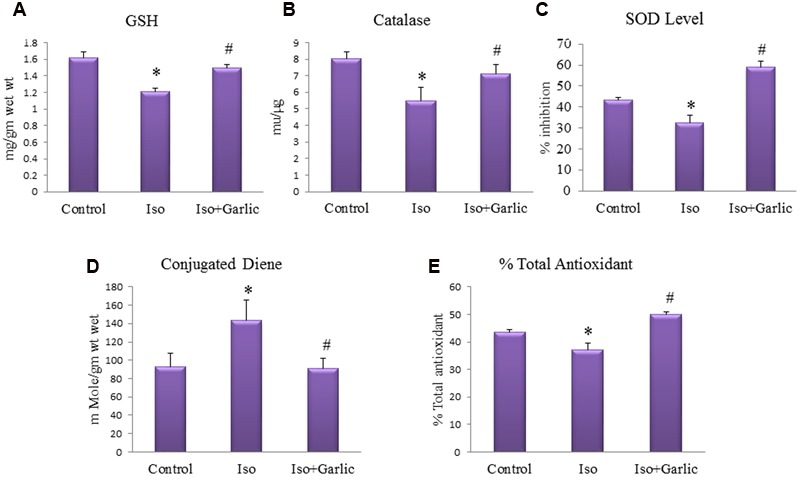
**Effect of garlic on endogenous antioxidants, conjugated dienes and total antioxidants. (A)** Myocardial GSH. **(B)** Myocardial catalase. **(C)** Myocardial super oxide dismutase. **(D)** Myocardial conjugated diene. **(E)** Myocardial total antioxidant capacity. (*N* = 6). Data were shown as mean ± SEM, ^∗^*p* < 0.05 vs. Control, ^#^*p* < 0.05 vs. Iso.

A significant (*p* < 0.05) decrease in myocardial GSH level, catalase and SOD activity and total antioxidant capacity was observed in Iso group compared to control group. However, a significant (*p* < 0.05) increase in these myocardial enzymes activity was observed in Iso+garlic group compared to Iso group (**Figures 3A–C,E**).

A significant (*p* < 0.05) increase in myocardial conjugated diene levels was observed in Iso group compared to control group. However, there was significant (*p* < 0.05) decrease in myocardial conjugated diene levels observed in Iso+garlic group compared to Iso group (**Figure 3D**).

### Garlic Treatment Enhanced Mitochondrial Enzymes Activity in Hypertrophy Heart

**FIGURE 4:**
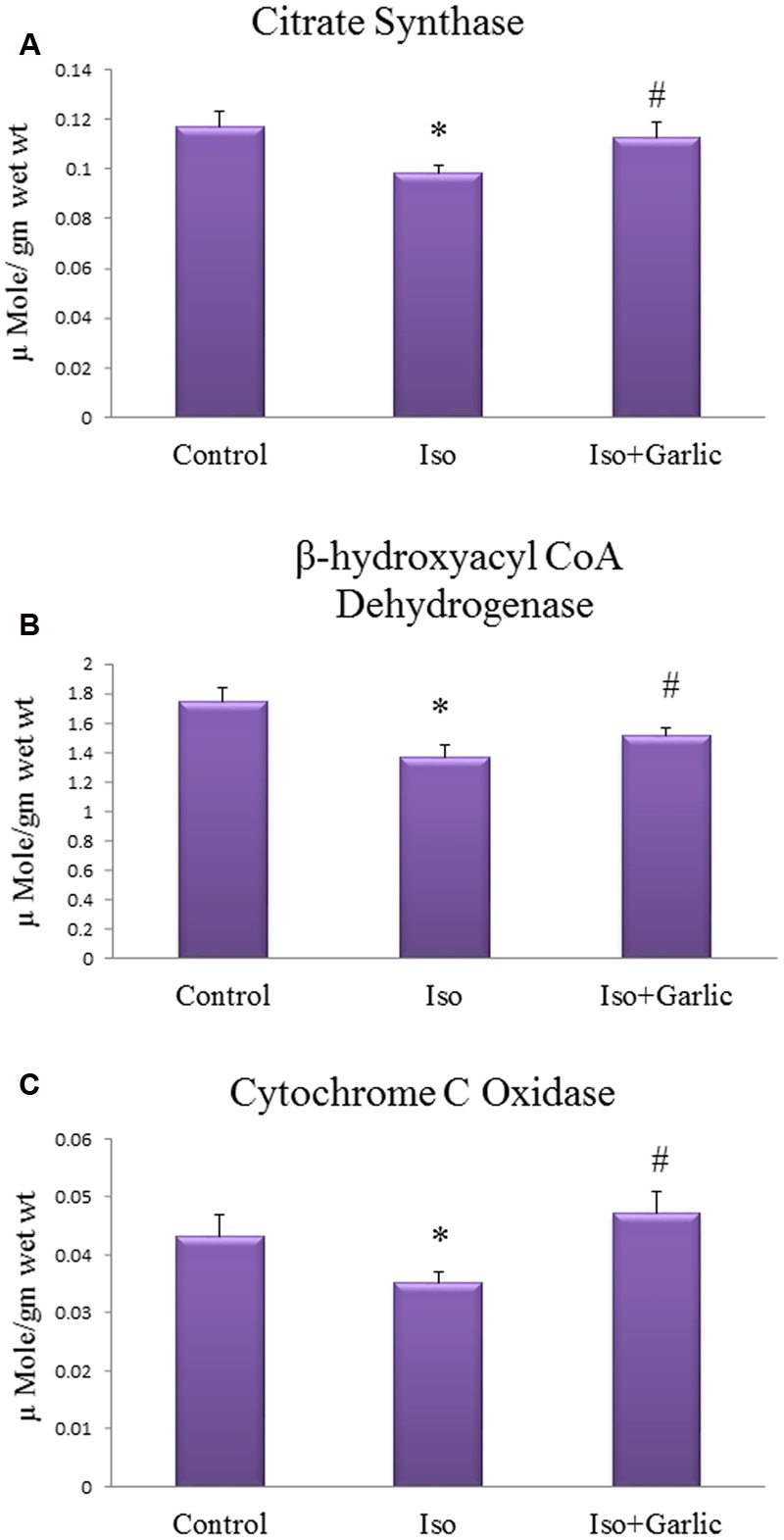
**Effect of garlic on mitochondrial enzyme activity. (A)** Citrate synthase activity **(B)** β-hydroxylacyl CoA dehydrogenase **(C)** Cytochrome C oxidase activity. (*N* = 6). Data were shown as mean ± SEM, ^∗^*p* < 0.05 vs. Control, ^#^*p* < 0.05 vs. Iso.

Mitochondrial enzymes in the heart are affected during hypertrophy and heart failure condition. Citrate synthase, β hydroxyacyl CoA dehydrogenase and cytochrome C oxidase are key mitochondrial enzymes involved in the Krebs cycle, β-oxidation, and electron transport chain activities, respectively. A significant (*p* < 0.05) decrease in these enzyme activities was observed in Iso group compared to control group. However, a significant (*p* < 0.05) increase in the activity of all those enzymes was observed in Iso+garlic group compared to Iso group (**Figures 4A–C**).

### Garlic Treatment Reduced Collagen Gene Expression and Normalized Histopathological Changes in Hypertrophy Heart

**FIGURE 5:**
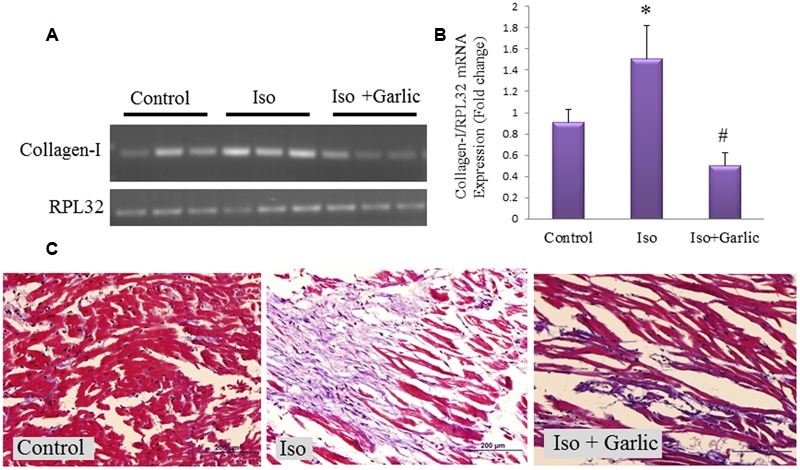
**Effect of garlic on collagen-I expression and myocardial injury after Iso administration. (A)** Myocardial collagen-I mRNA expression. **(B)** Bar graph of collagen-I expression. **(C)** Fibrosis as observed by Masson’s Trichrome stain (scale bar 200 μm). Data were shown as mean ± SEM (N = 3), ^∗^*p* < 0.05 vs. Control, ^#^*p* < 0.05 vs. Iso.

Collagen-I mRNA expression is a marker of myocardial fibrosis. Collagen-I is the most abundant among all types of collagens. There was significant (*p* < 0.05) increase in collagen-I gene expression in Iso group compared to control group. However, a significant (*p* < 0.05) decrease in collagen-I gene expression was observed in Iso+garlic group compared to Iso group (**Figures 5A,B**). We have also looked gene expression of three apoptotic markers, i.e., Bax, Bad, and caspase 3 in H9C2 cells after Iso treatment and co-treatment with garlic and sulfur metabolites. There was no significant alteration of these genes between control and iso group (Supplementary Figure S2).

We observed myocardial fibrosis in Iso group after staining with Masson’s trichrome. While light micrograph of control heart showed normal architecture and no fibrosis, hypertrophic heart (Iso) showed massive fibrosis in cardiac muscle along with infiltration of acute and chronic inflammatory cells. However, our finding showed that fibrosis, as well as myocardial injury, was reduced after garlic treatment (Iso+garlic group) (**Figure 5C**).

### Allyl methyl Sulfide (AMS), Allyl Methyl Sulfoxide (AMSO) and Allyl Methyl Sulfone AMSO2 Are Most Abundant Garlic Metabolites Present in Serum after Oral Administration of Garlic Homogenate

**FIGURE 6:**
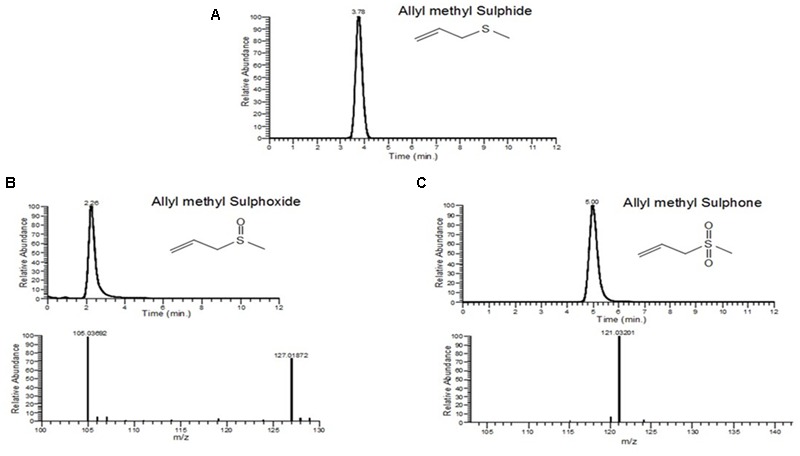
**U-HPLC/APCI-MS-EIC of (A)** allyl methyl sulfide (*m/z* 89, Rt-3.78 min). **(B)** Allyl methyl sulphoxide (*m/z* 105,Rt = 2.26 min). **(C)** Allyl methyl sulfone(*m/z* 121,Rt = 5.00 min).

The metabolites of garlic homogenate were detected after oral administration of raw garlic in rats. The identified metabolites of garlic in rat plasma are AMS, AMSO and AMSO2. **Figure 6** shows the U-HPLC/APCI-HRMS-EIC metabolites of garlic homogenate formed in the plasma. To elucidate the structures of the metabolites, the [M+H]^+^ ions of allyl methyl sulphoxide and AMSO2 by using U-HPLC-APCI-HRMS experiments in combination with accurate mass measurements was studied. Three compounds were identified by comparing their retention times and or mass spectra with those of synthesized standard compounds (**Figures 6A–C**; **Table 2**).

**Table 2:** Elemental compositions for garlic components obtained after LC-MS study.

Metabolites	Formula	Observe mass (Da)	Calculated mass (Da)	Error (ppm)
Allyl methyl sulfide	C_4_H_9_S	89.04277	89.04250	-3.03
Allyl methyl sulphoxide	C_4_H_9_SO	105.03692	105.03741	4.67
				
Allyl methyl sulfone	C_4_H_9_SO_2_	121.03201	121.03233	2.64

### Garlic and Its Metabolites Attenuated Cell Size and Intracellular Calcium Level in H9C2 Cells

**FIGURE 7:**
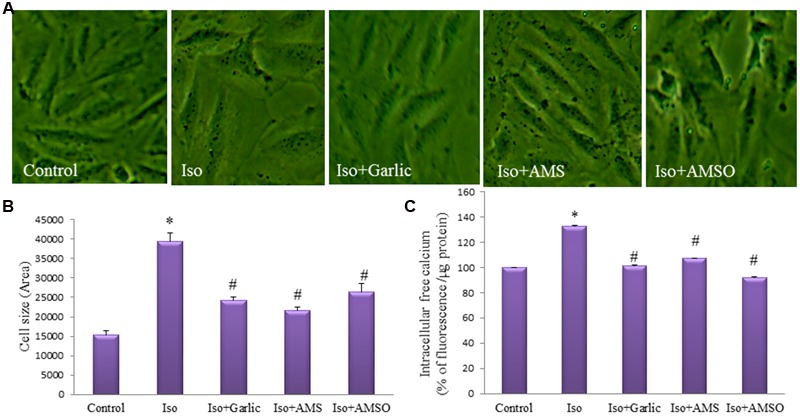
**Effect of garlic and its metabolites on hypertrophy of H9C2 cells. (A)** Images of H9C2 cells to measure cell size. **(B)** Bar diagram of cell sizes. **(C)** Intracellular calcium level in H9C2 cells. (*N* = 20). Data were shown as mean ± SEM, ^∗^*p* < 0.05 vs. Control, ^#^*p* < 0.05 vs. Iso.

In hypertrophy, the rate of protein synthesis generally increases. Therefore, the cross-sectional area of cardiac cells, i.e., cell size is a marker of cardiac hypertrophy. There was a significant (*p* < 0.01) increase in cell size in Iso group in comparison to control group. However, a significant (*p* < 0.01) decrease in cell size was observed in garlic and its metabolites treated groups compared to Iso group (**Figures 7A,B**). Similarly, intracellular calcium concentration was increased significantly (*p* < 0.05) in Iso group and decreased (*p* < 0.05) after garlic and its metabolites treatment (**Figure 7C**).

### Improvement of Na^+^/K^+^-ATPase in Hypertrophy Heart and H9C2 Cells after Garlic and Its Metabolites Treatment

**FIGURE 8:**
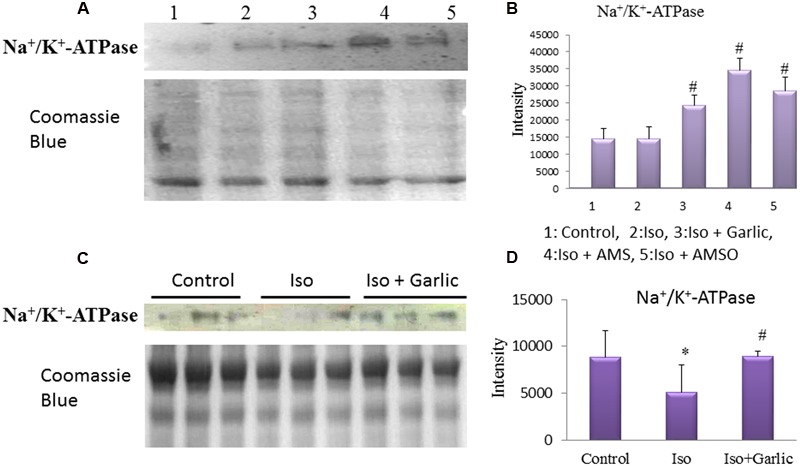
**Effect of garlic and its metabolites on Na^+^/K^+^-ATPase expression. (A,B)** Western blot and bar graph of Na^+^/K^+^-ATPase from H9C2 cells. **(C,D)** Western blot and bar graph of Na^+^/K^+^-ATPase from rat heart. (Gels and blots were cropped and run under same experimental conditions). (*N* = 3). Data were shown as mean ± SEM, ^∗^*p* < 0.05 vs. Control, ^#^*p* < 0.05 vs. Iso (2).

Na^+^/K^+^-ATPase is an important enzyme of heart contraction. Prolong inhibition of this enzyme creates hypertrophy and heart failure. To find the effect of garlic and its metabolites on Na^+^/K^+^-ATPase protein levels, we did immunoblotting from heart and H9C2 cell. No change in Na^+^/K^+^-ATPase protein expression levels was observed in H9C2 cells treated with Iso compared to control cells. However, a significant (*p* < 0.05) increase in Na^+^/K^+^-ATPase levels was observed in garlic and two garlic metabolites treated groups (**Figures 8A,B**).

A significant (*p* < 0.05) decrease in Na^+^/K^+^-ATPase protein levels was observed in Iso group compared to control group. However, a significant (*p* < 0.05) increase Na^+^/K^+^-ATPase expression was observed in Iso+garlic group compared to Iso group (**Figures 8C,D**).

### Garlic and Its Metabolites Failed to Show Any Anti-Hypertrophic Effect in H9C2 Cells in Presence of Na^+^/K^+^-ATPase Inhibitor

To confirm whether garlic and its metabolites showed their anti-hypertrophic effect through Na^+^/K^+^-ATPase, we treated H9C2 cells with Iso in presence and absence of digoxin, a Na^+^/K^+^-ATPase inhibitor. There was an increase (*p* < 0.05) of cell size in iso group and iso+digoxin groups. However, garlic as well as two metabolites (AMS and AMSO) failed to attenuate the Iso-induced hypertrophic effect in presence of digoxin (Supplementary Figure S1).

## Discussion

The present study revealed that garlic protects against cardiac hypertrophy by inhibiting oxidative stress and improving mitochondrial efficiency. Our finding supported that garlic could be a promising nutritional agent for therapies against cardiac hypertrophy and remodeling. Stimulation of the β-adrenoceptors by Iso is a well-known animal model of cardiac hypertrophy without systolic hypertension (Galindo et al., 2009). Iso induces myocardial hypertrophy as well as injury in rats through several mechanisms, including oxidative stress, ROS generation, an increase in collagen deposition, intracellular calcium overload, inflammation and exhaustion of high-energy phosphates through reduction of mitochondrial efficiency (Sharma et al., 2010). Excess free radical production in the heart after Iso may cause necrosis, loss of contractile tissue, fibrosis and severe arrhythmias. All those changes in heart ultimately induce cardiac hypertrophy and remodeling with dilatation over time in rats.

There are several natural or nutritional agents that were used previously to reduce oxidative stress in heart and improved cardiac health. Among them, garlic attracted more scientific attention for its beneficial effect against cardiovascular disorder (Banerjee and Maulik, 2002). Although, attenuation of cardiac hypertrophy after allicin administration was observed (Liu et al., 2010), the active component responsible for this beneficial effect was not clear. In the present study, we administered raw garlic homogenate, rich in allicin, in rats and identified the active garlic metabolites through LC/APCI/HRMS study. Our data showed that raw garlic was effective to reduce cardiac hypertrophy and associated remodeling in the heart through those active metabolites.

The previous study reported that oxidative stress plays an important role to develop cardiac hypertrophy and its progression to heart failure. Sustained β-AR activation was shown to promote oxidative stress in cardiac myocytes as evidenced by increased lipid peroxidation as well as decreased endogenous antioxidant (Rehsia and Dhalla, 2010). Similarly, our data also showed a significant increase in lipid peroxidation as observed by a higher level of conjugated dienes and decrease in endogenous antioxidants like SOD, catalase and GSH in rat heart after Iso treatment. Increased oxidative stress as observed in our study might be due to overproduction of free radical and ROS. Alteration of mitochondrial efficiency during disease condition may enhance ROS generation. Mitochondria, which occupy a large portion of the myocyte play a crucial role to supply energy to the heart. Scientific evidence suggests that mitochondria play an important role in the progression and development of heart disease (Ide et al., 1999). The scientific literature showed that alterations in mitochondrial function are involved in the decompensation of cardiac hypertrophy. In the present study, we focused on changes in mitochondrial enzymes activities and alteration of oxidative stress during hypertrophy. Our data indicated that mitochondrial enzymes like citrate synthase, beta hydroxyl acyl CoA dehydrogenase and cytochrome c oxidase were decreased in Iso-treated heart. However, all those three enzymes increased significantly after garlic administration.

Increased myocardial fibrosis and collagen deposition is a hallmark of cardiac hypertrophy (Ho et al., 2010). It is mostly due to secondary response to the pathophysiological remodeling of long-standing disease. In the present study, we observed a significant increase in collagen-I mRNA expression and fibrosis in Iso-treated heart. However, administration of garlic normalized the higher collagen-I expression and fibrosis in Iso-treated heart. Along with collagen synthesis, increased myocytes injury was observed in Iso-treated heart and decreased significantly after garlic treatment.

Gaseous molecules were determined in the present study to understand the protective effect of garlic on cardiac hypertrophy. Nitric oxide signaling declines during cardiac hypertrophy (Zhang et al., 2012). Inhibition of nitric oxide synthesis increases cardiac hypertrophy and fibrosis in rat’s subjected to aerobic training (Souza et al., 2007). Similar to nitric oxide, hydrogen sulfide also plays an important role in cardiac hypertrophy. Hydrogen sulfide attenuates cardiac hypertrophy and fibrosis induced by abdominal aortic coarctation in rats (Huang et al., 2012). Plasma high hydrogen sulfide concentration may enhance cardioprotection (Wang et al., 2011). Similar to previous studies, we also observed a significant reduction of myocardial NO and H_2_S level. However, raw garlic administration normalized those two important gaseous molecules.

Although we have observed a significant reduction of cardiac hypertrophy, the molecular mechanism and the active metabolites of garlic responsible for this beneficial effect is not well-understood. Allicin, the main active ingredients of garlic homogenate, was not present in the blood after oral consumption of raw garlic homogenate. Therefore, it was very important to identify the active metabolites of garlic *in vivo*. We identified three sulfur compounds by LC-MS study from rat plasma after oral administration of raw garlic. These three compounds are AMS and AMSO, AMSO2. To confirm the anti-hypertrophic effect of those metabolites, we treated H9C2 cells (cardiomyocytes) with raw garlic and two of its active metabolites, AMS and AMSO. Several research papers also used this H9C2 cells as one of the cell lines to validate many *in vivo* data. Although there are limitations of this cell line for certain cardiac activity especially contraction and relaxation, this cell was extensively used for inducing hypertrophy to look cellular signaling pathways (Jeong et al., 2009). Our data confirmed that raw garlic homogenate as well as two garlic metabolites, AMS and AMSO significantly decreased the cell size.

The molecular mechanism by which garlic and its metabolites reduced cardiac hypertrophy might be several. Our data found that garlic and its two metabolites can enhance Na^+^/K^+^-ATPase level in heart and H9C2 cells. Prolong inhibition of Na^+^/K^+^-ATPase or reduction of Na^+^/K^+^-ATPase levels in the heart results in progressive accumulation of intracellular Na^+^ ion which indirectly results in intracellular Ca^2+^ accumulation. Na^+^/K^+^ pump efficiently couple to Na^+^/Ca^2+^ exchanger (NCX) to maintain Ca^2+^ ion regulation. (Shattock et al., 2015). Low Na^+^/K^+^-ATPase levels or activity promote Ca^2+^ influx via NCX leading to Ca^2+^ accumulation which can accelerate myocardial necrosis, contractile failure and also trigger arrhythmias. Calcium overload is one of the mechanisms of Iso-induced cardiac hypertrophy and heart failure (Garg and Khanna, 2014) and also observed in our Iso treated H9C2 cells. Specifically, in cardiac hypertrophy, many studies have shown that Na^+^/K^+^ pump function, and/or expression, is reduced. The Na^+^/K^+^-ATPase activity in rat sarcolemma after Iso-induced myocarditis decreases by 42% (Osadchaia et al., 1990). Thus, a correlation between the decrease in heart function and the decrease in Na^+^/K^+^-ATPase concentration exists (Schwinger et al., 2003). In a failure heart, ventricular weight was markedly increased along with the significant reduction of Na^+^/K^+^-ATPase activity (Yazaki and Fujir, 1972). In the present study, we have observed a significant decrease of Na^+^/K^+^-ATPase protein level in hypertrophy heart. Therefore, it could be possible that Ca^+2^ overload due to lower Na^+^/K^+^-ATPase level might be the major contributor for cardiac hypertrophy in our rat model. However, garlic treatment normalized the Na^+^/K^+^-ATPase level in Iso-treated hypertrophy heart and thus may reduce the Ca^+2^ overload. Our data confirmed that garlic and both metabolites AMS and AMSO have a property to increase Na^+^/K^+^-ATPase protein level and decrease intracellular calcium level, and thus have ability to attenuate cardiac hypertrophy. Reduction of cardiac hypertrophy by garlic and its metabolites is summarized in the graphical abstract.

## Conclusion

Garlic and its identified metabolites could prevent Iso-induced hypertrophic growth in rat heart and H9C2 cells though partially increased Na^+^/K^+^-ATPase level. Identified and characterized garlic metabolites viz. AMS and AMSO could be developed as potential drug molecules against cardiac hypertrophy.

## Author Contributions

TK and SB designed the research. TK, AD, SM, RB, RS, and SB performed experiments and analyzed data. TK and SB wrote the manuscript and had full access to all the data in the study and take responsibility for the integrity of the data and the accuracy of the data analysis.

## Conflict of Interest Statement

The authors declare that the research was conducted in the absence of any commercial or financial relationships that could be construed as a potential conflict of interest.

## Abbreviations

AMS: allyl methyl sulfide
AMSO: allyl methyl sulfoxide
AMSO2: allyl methyl sulfone
DPPH: 2,2-Diphenylpicrylhydrazyl
GSH: Glutathion
H&E: hematoxylin and eosin
Iso: Isoproterenol
LDH: lactate dehydrogenase
NCX: Na^+^/Ca^2+^ exchanger
PCR: Polymerase chain reaction
RPL32: Ribosomal protein L32
SGOT: serum glutamic oxaloacetic transaminase
SOD: superoxide dismutase
β-MHC: β-Myosin Heavy Chain
