# Supplementary material for: Novel Sulfur Metabolites of Garlic Attenuate Cardiac Hypertrophy and Remodeling through Induction of Na+/K+-ATPase Expression
Source: Front Pharmacol. 2017 Jan 30;8:18. doi: 10.3389/fphar.2017.00018 (PMC5276815; doi:10.3389/fphar.2017.00018)
Supplement: Supplementary file 1 [file Data_Sheet_1.docx]

**Supplementary Data**

**Determination of Cell surface area**

Protocol for measurement of cell surface area was followed as described by Ioannis et al., 2013 after slight modification. Briefly, following treatments, H9C2 cells in six-well plates were washed thrice with phosphate-buffered saline (PBS), then they were ﬁxed with 1ml of ice-cold methanol for10 min and stained with 0.1% crystal violet for15 min. Excesses of dye in the plates was washed with PBS 4-5 times. Plates were left to dry overnight, and cell images were acquired using a Nikon microscope (Eclipse TS 100) with attached digital camera and 10× lens. Ten random photographs were taken from each sample group, and at least 100 individual cells were examined in each group. Cell size was analyzed using Scion Image Beta 4.02 software (Scion Corporation, USA, <http://www.scioncorp.com>).

**Results**


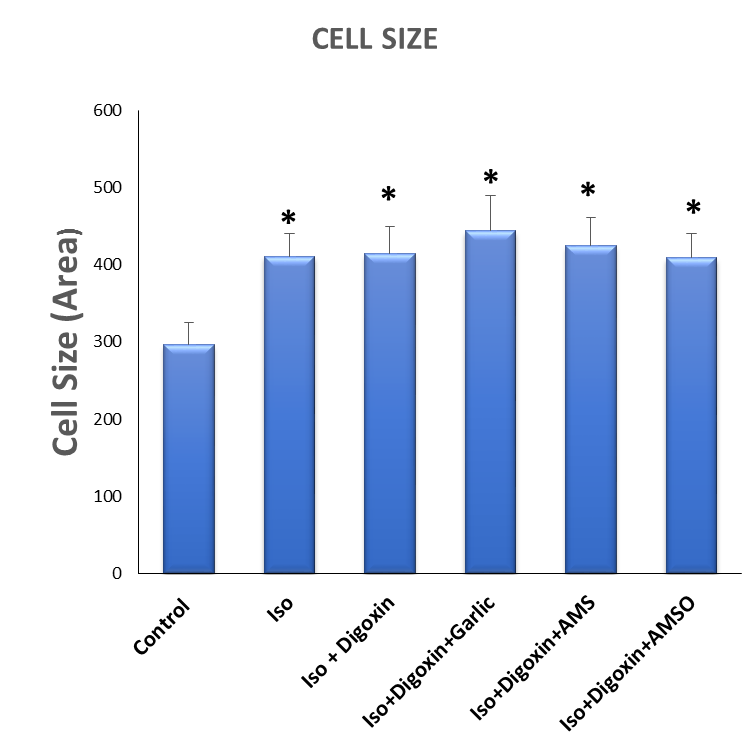

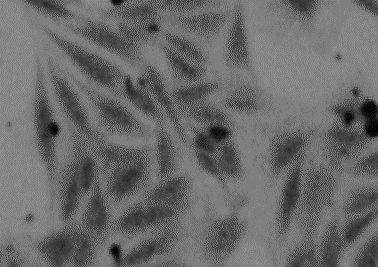

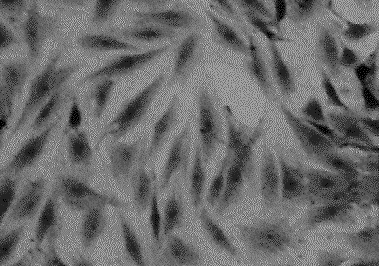

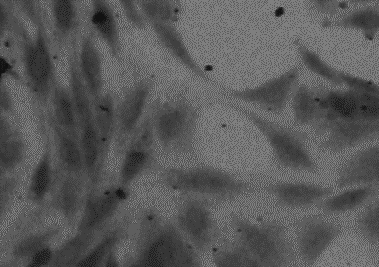

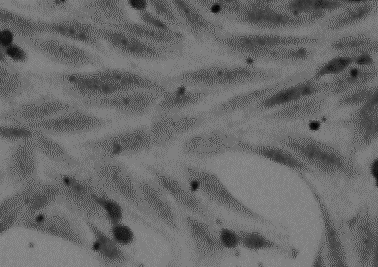

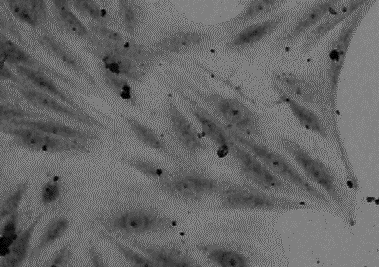

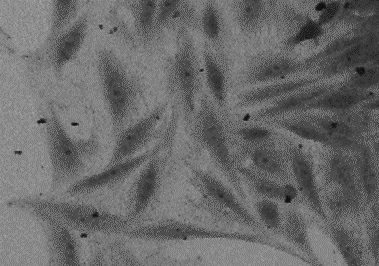


**Control**

**Iso + Digoxin + AMSO**

**Iso + Digoxin + AMS**

**Iso + Digoxin + Garlic**

**Iso**

**Iso + Digoxin**

(A)

(B)

**Fig S1:** Effect of Garlic and its metabolites on hypertrophy of H9C2 cells in presence of Na/K-ATPase inhibitor (Digoxin). **A]** Images of H9C2 cells to measure cell size. **B]** Bar graph of cell sizes. Data were shown as mean ± SEM (*N*=100), **p*<0.05 versus Control.


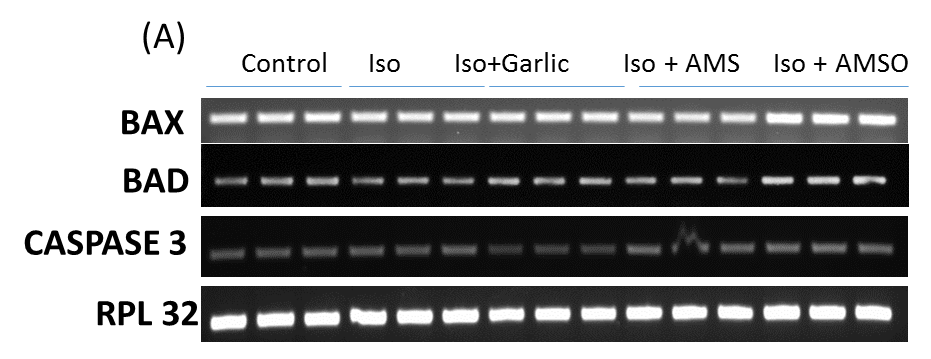


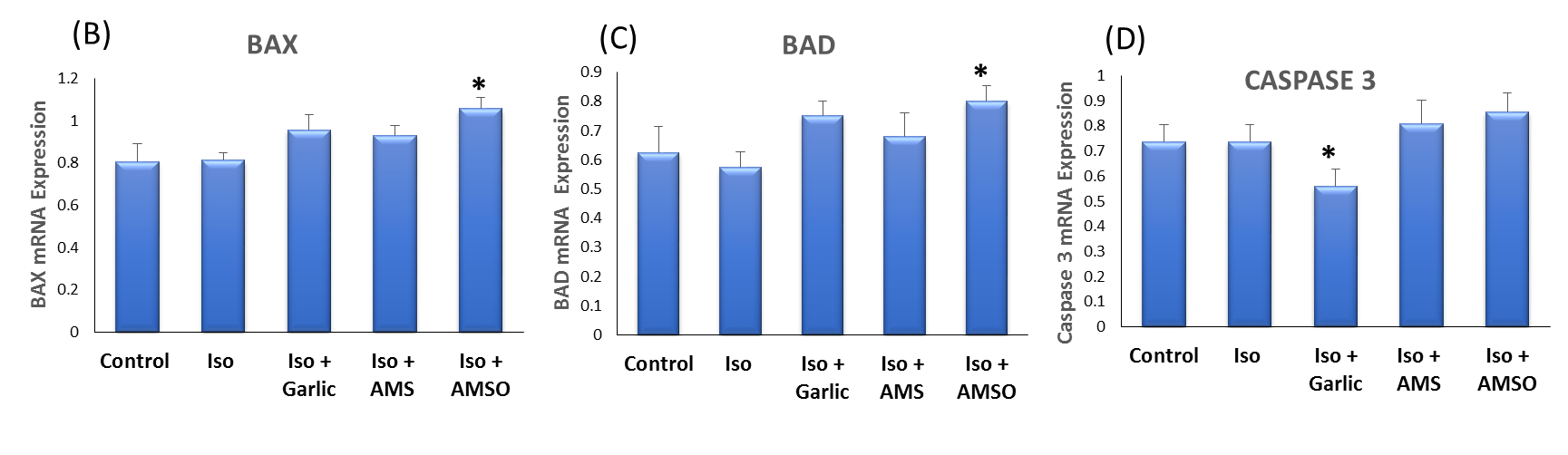


**Fig S2:** Effect of Garlic, AMS and AMSO on apoptosis gene expression in H9C2 cells following Isoproterenol treatment. **A]** Apoptotic markers BAX, BAD and Caspase 3. **B]** Bar graph for BAX mRNA expression. **C]** Bar graph for BAD mRNA expression. **D]** Bar graph for Caspase 3 mRNA expression. Data were shown as mean ± SEM (*N*=3), **p*<0.05 versus Control.

**Reference**

Ioannis, A., Anthula, K., Ioannis, T., Alexandros, K., Mihalis, P., Antigone, L., et al. (2013).Silibinin protects H9c2 cardiac cells from oxidative stress and inhibits phenylephrine-induced hypertrophy: potential mechanisms. *Journal of Nutritional Biochemistry*. 24,586–594.DOI:10.1016/j.jnutbio.2012.02.009
